# The Two Facing Square Flaps Method for Release of Anterior and Posterior Axillary Line Burn Contractures

**DOI:** 10.3390/ebj4040034

**Published:** 2023-10-04

**Authors:** Whitney Laurel Quong, Rei Ogawa

**Affiliations:** 1 Department of Plastic, Reconstructive and Aesthetic Surgery, Nippon Medical School, Tokyo 113-8603, Japan; whitney.quong@mail.utoronto.ca; 2 Division of Plastic, Reconstructive & Aesthetic Surgery, University of Toronto, Toronto, ON M5T 1P5, Canada

**Keywords:** axilla, axillary contracture, axillary reconstruction, burn reconstruction, local flap, square flap, square flaps method

## Abstract

With improved burn outcomes and survival rates, the focus of management in large burns has shifted from merely survival towards optimizing form and function for the burn survivor. Due to its unique structural features and functional demands however, the axilla is prone to contracture formation, and remains complex to reconstruct. Where contractures involve both the posterior and anterior axillary lines, the two facing square flaps method is a suitable choice for a wide range of patients. The flap design is flexible, and is relatively safe with a sufficient blood supply. Superior lengthening of approximately 3–4 times can be achieved, and is maintainable. In this surgical technique paper, we describe the strategy of the two facing square flaps, and present two patterns of its application, with representative cases of the local flap method.

## 1. Introduction

It has been estimated that approximately 2.5 million people seek medical care annually for acute burns in the United States [1]. Yet, while burn survival and outcomes are continually improving due to advancements in evidence-based practice, certain challenges persist for burn survivors and their reconstructive surgeons. For those who suffer severe burns of the trunk and upper arm, contractures of the axilla remain a difficult surgical problem. 

As it is paired with the most mobile joint in the human body, has a complex surface contour, and is subject to significant tensile forces, the axilla is prone to skin graft loss and resulting contractures. Release of these contractures, and restoration of mobility and function requires considered selection of the appropriate surgical technique. An initial step in approaching these cases is to define the specific axillary subunits which are involved in the contracture. A commonly applied classification system for doing so was defined by Kurtzman [2], and involves three main categories:

- Type IA: injuries involving the anterior axillary fold;
- Type IB: injuries involving the posterior axillary fold;
- Type II: injuries involving both the anterior and posterior axillary folds;
- Type III: type II injuries plus axillary dome injuries.

In our 25-year retrospective review of axillary contractures in 2003, we further expanded on this classification system [3]:

1. Contractures within the axillary area
  - Type I: small, thin, flat contractures (a small part of the axilla is contracted, but the fat layer is conserved);
  - Type IIa: band contracture on the anterior axillary line;
  - Type IIb: band contracture on the posterior axillary line;
  - Type IIIa: contractures on both the anterior and posterior line with no contractures between the lines;
  - Type IIIb: contractures on both the anterior and posterior line with contractures between the lines.
2. Contractures extending outside the axillary area
  - Type IVa: contractures extending to both the chest and upper arm with no contractures on the back;
  - Type IVb: contractures extending to both the back and upper arm with no contractures on the chest;
  - Type IVc: contractures extending to both the chest and back with no contractures on the upper arm;
  - Type V: extensive contractures except Type IV.

Compared with Kurtzman’s proposal, our classification system makes a preliminary distinction between those contractures which are completely within the axilla, and those which extend beyond it. We also further expanded certain categories, to account for nuances relevant to the reconstructive surgeon. In particular, our classification system facilitates selection of the appropriate surgical technique on the reconstructive ladder. Here we propose the two facing square flaps technique for Type III contractures involving both the anterior and posterior axillary lines. However, the surgical approach may be effective even where the contracture extends beyond the axilla in the appropriate patient. We discuss patient selection, application of this specific technique, and advantages of this strategy. 

## 2. Surgical Technique

Prior to applying the two facing square flaps method for axillary contracture release, the patient must be carefully selected. The most important factor to consider is the scar morphology and axillary subunit involvement. This technique is most appropriate in those who have medium depth, medium-sized band-like contractures involving both the anterior and posterior axillary lines, and where the scar does not involve the central region delimited by these contractures (Type IIIa). 

This technique is contraindicated for more extensive planar scar contractures, where it is necessary to completely remove the scar, and resurface the area with a larger transposition flap or, in severe cases, a free skin flap. The application of the square flap method is strictly for linear scar contractures or contractures where scar and normal skin are mixed. In patients who have fragile healed scars around the shoulder girdle and adjacent chest (Type IV, or V), if the axilla is surrounded by thick scars, it may be necessary to bring a large free flap or regional flap from another area to recruit normal and more mobile tissues. In such cases, this technique alone may not be sufficient. However, if the surrounding scars are less severe, the contracture release effect might be diminished, but this technique may still be applicable, and a flap can be designed over the scar. In terms of holistic patient characteristics, unlike free flap procedures which demand a certain level of patient fitness, as a local flap option, the two facing square flap technique can be applied over a broad range of patients. This technique is therefore a favourable choice in the elderly and those with pre-existing comorbidities. Additionally, this reconstructive strategy may be particularly attractive in low-resource settings where free flap procedures are not feasible [4]. 

**Figure 1:**
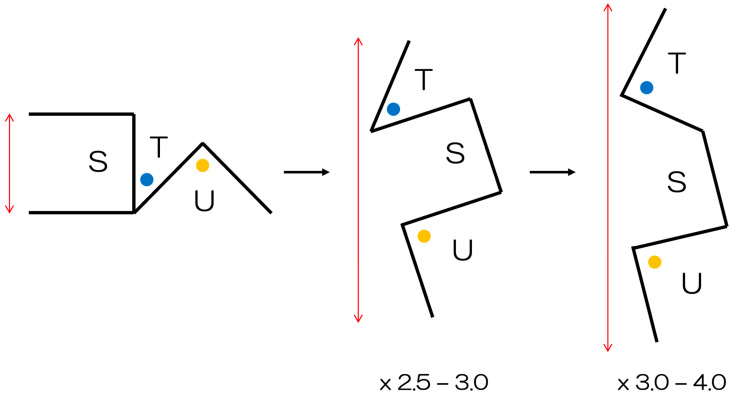
The square flap method (adapted from Huang et al. [5]). The local flap incorporates a square flap (S-flap), a triangular flap with a tip ideally forming a 45° angle, and a second triangular flap with a 90° tip (U-flap). All flap edges are of equal length. With this method, approximately 3–4 times lengthening is achievable.

The fundamental design of the square flap method is shown in Figure 1. The flaps are named S-flap, T-flap, and U-flap. All sides of the flaps are of equal length. The T-flap tip was originally designed to ideally be 45°, while the U-flap was described to be a right angle. However, depending on the morphology and location of the surrounding scars it is possible to modify the design, such that the triangular flaps are as 45 and 45°, or 45 and 60°. Angles between 45° and 90° can efficiently maintain blood flow at the tip of the skin flap, effectively relieve contracture, and allow for donor closing. There is significant flexibility of design in this method. 

**Figure 2:**
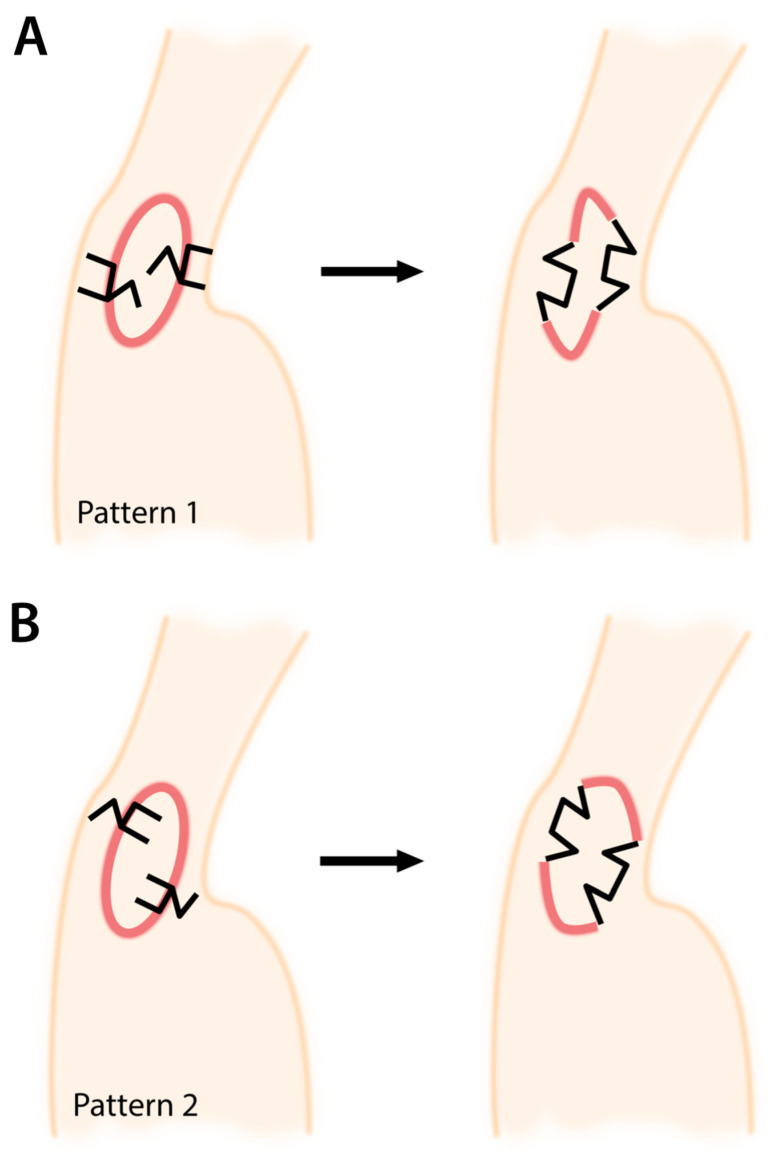
The two facing square flaps method for axillary burn contracture release. Two patterns are shown: (**A**) Pattern 1, and (**B**) Pattern 2. When planning the two facing square flaps, the common limbs between the square flaps and T-flaps lies along the contracture, with one square on the posterior, and one on the anterior axillary line. Both S-flaps lie either within the circular contracture, or outside of the contracture.

Before designing the two facing square flaps for axillary contracture release, the contracture itself should first be noted. The contractures most often form an ovoid/circular shape, with the longitudinal axis oriented in a superior-inferior (cephalad-caudal) direction (Figure 2). The common limb between the square flap (S-flap) and the adjacent triangle (T-flap) should then be drawn along the contracture forming this oval. For each square flap, the S-flap should lie on the same side of the oval – both either outside (pattern 1), or inside (pattern 2) of it. One square flap should be placed superiorly, and one inferiorly, on opposite sides of the contracture (one along the anterior, and one along the posterior axillary line). Ultimately however, the orientation of the flap should be considered on a case-by-case basis depending on the surrounding axillary scar. 

Regarding the choice between the patterns, there are no definitive indications for either—it is dependent on the clinical case and the associated anatomy. The effects obtained are equivalent. However, by designing the square flap on normal skin, the postoperative rectangular flap becomes a trapezoid shape with scar maturation, effectively relieving the scar contracture and tension. Meanwhile, a triangular flap placed within the scar can lead to poor blood flow at the tip, and risk necrosis. It is necessary to customize the use of two facing square flaps to the particular scar anatomy.

After subcutaneous infiltration with local anaesthesia and adrenaline, the skin is incised with a 15 blade down to the subcutaneous fat. The flaps are then mobilized to assess the degree and adequacy of release. A moderate amount of undermining may be necessary. Hemostasis is then achieved with electrocautery. The subcutaneous/superficial fascial layer is approximated with 3-0 PDS II, the dermal layer with 4-0 PDS II, and the superficial layer with 6-0 Ethilon. Sutures are removed 7–10 days after surgery, and patients undergo tape fixation for 3 months postoperatively. Until suture removal, patients are advised against excessive arm movement, and we particularly note that patients are to be cautious when changing their clothes. A physiotherapy/rehabilitation protocol transitioning from passive range of motion to active range with strengthening is necessary.

## 3. Illustrative Cases

### 3.1. Case 1

**Figure 3:**
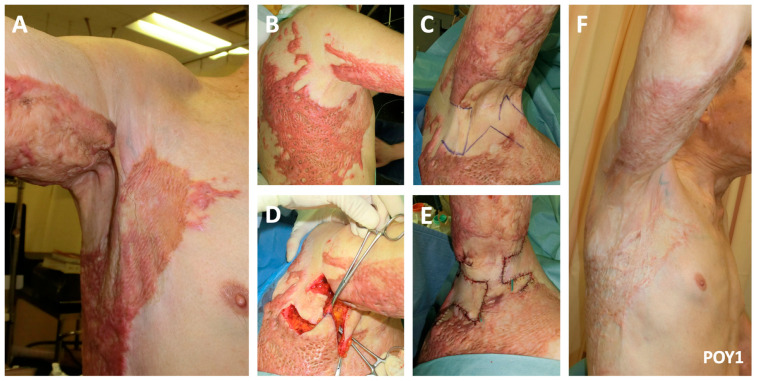
A 74-year-old man underwent the two facing square flaps method for axillary contracture release (Pattern 1). A significant contracture and restriction of both active (**A**) and active (**B**) shoulder range is noted preoperatively. (**C**) Pattern 1 of the two facing square flaps is designed. The flaps are incised, and the (**D**) skin advancement is noted. (**E**) The immediate postoperative result is noted. (**F**) One-year postoperatively, adequate range of motion is achieved.

A 74-year-old man one year post flame burn causing a right-sided axillary contracture involving both the anterior and posterior axillary lines (Type IIIa) presented to our clinic with limited shoulder abduction (Figure 3). Pattern 1 of the two facing square flaps method was performed. One-year post reconstruction, near-complete shoulder abduction was achievable. 

### 3.2. Case 2

**Figure 4:**
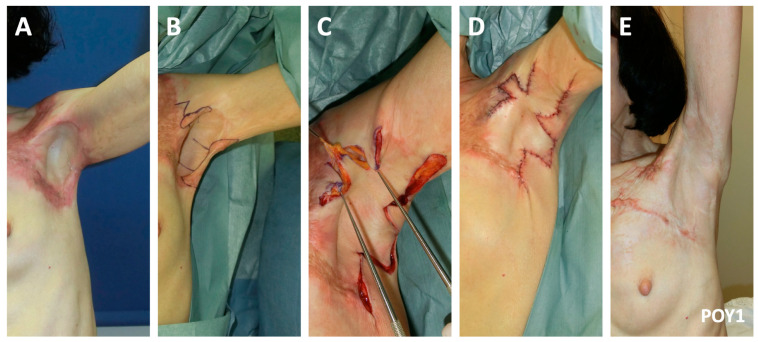
One year post burn, a 73-year old woman with limited shoulder range underwent Pattern 2 of the two facing square flaps method for axillary contracture release. (**A**) The axillary contracture involving both the anterior and posterior lines was evident preoperatively (**A**). (**B**) The flaps were designed, and (**C**) advanced. A portion of the scar was also excised to facilitate flap advancement and reorientation. (**D**) The immediate postoperative result demonstrated improved shoulder range. (**E**) Near complete shoulder abduction was maintained at the one-year postoperative follow-up.

One-year post flame burn, a 73-year-old woman presented with limited shoulder abduction secondary to contracture of the anterior and posterior axillary lines (Type IIIa; Figure 4). She underwent release of the contracture using Pattern 2 of the two facing square flaps method. One-year postoperatively, she maintained full shoulder range. 

## 4. Discussion

Selecting the most appropriate reconstructive method to release an axillary contracture is a primary factor in determining the ultimate outcome. That is, success is dependent upon matching the clinical case with the surgical strategy. Yet, navigating the reconstructive methods for axillary contracture release can be challenging, given the various methods that have been proposed [3,6,7,8]. To streamline this process, we have previously outlined that the size, depth, location, and shape of the contracture [3] are the most important features of the contracture to consider [3,5]. Only where the scar is small in size, and shallow (where the fat layer is conserved), should skin grafts be selected [3]. Where the scar tissues do not glide however, a flap-based approach (whether local or free) must be utilized. For single band contractures along the anterior or posterior axillary lines (Types IIa and IIb), a scar lengthening procedure such as a Z-plasty, five-flap method, or single square flap method can be considered. However, if the bands involve both axillary lines, or is of high grade and the contracted skin forms a web, a local flap, or the two facing square flaps method should be considered.

First described in 1987 by Hyakusoku, the square flap method comprises a square flap (S-flap), one acutely-angled triangular flap (T-flap), and another right-angled triangular flap (U-flap) [9]. The details and case analyses of the (single) square flap method have been thoroughly described in previous papers [5,10,11]. In essence however, the square flap method is a 3-flap Z-plasty that is composed of a square advancement flap along with two triangular transposition flaps [5]. Compared with a similar method described by Limberg [12], and other Z-plasty methods, the square flap method achieves better lengthening [5], as one of the triangular flaps is a right angle. The square flap method also has additional anatomical and physiological advantages, including low physiological tension (and therefore less deformity of the surrounding tissues), and a greater blood supply [5]. It is specifically worthwhile mentioning, that although techniques such as multiple and 5-flap Z-plasties are common in this application [13,14], these methods often result in the creation of many small acute-angled triangular flaps. Particularly in already scarred burn skin, where vascular status is compromised at baseline, the tips of these triangular flaps are therefore prone to necrosis. Our square flap method however, which designs the square flap on the more pliable available skin, is very safe in terms of blood flow by comparison, and can effectively release tension. Furthermore, the square flap method also allows for significant advancement of the square flap, while avoiding suture lines running parallel to the direction of lengthening [9]. 

Modifications to each square flap of the two facing square flaps method are also possible, and may be necessary to optimize the result based on the clinical case. In fact, this flexibility of design is a significant benefit of the method. Specifically, depending on the surrounding tissues, and desired vector of lengthening, it is possible to increase the angle of the T-flap, while decreasing the angle of the U-flap. As long as the triangular flap tips are between 45 and 90°, blood flow can be maintained, and necrosis likely avoided. Altering the angles of these triangular transposition flaps essentially modifies where the square flap is advanced to. Deciding how to draw the square flap then therefore requires the surgeon to consider which healthy tissues are available to be advanced (the square portion), and in which direction would it be most advantageous to advance these tissues.

We, along with others, have previously described application of the single square flap method for axillary release in the context of Type IIa and IIb contractures [3,5,15]. When applying two facing square flaps method however, one on the anterior, and one on the posterior axillary line, the same basic flap can be used to treat Type IIIa contractures of medium size and depth. It is worthwhile to note here, that although we have described two patterns of this technique, there are multiple orientations of the two facing square flaps that are possible. It is most important that the square flap be designed on normal skin such that it can be advanced advantageously. Regardless of how they are drawn, if performed with our guidelines in mind, with the two facing square flaps method, the circular/ovoid scar is broken up, resulting in a zig-zag suture line. This reorientation of the scar is particularly beneficial, as it reduces the incidence axillary contracture reformation. In fact, recurrence of axillary contractures is a considerable risk with reconstructive procedures in this area [16,17,18,19]. With lengthening of approximately 3–4 times achieved by half a year postoperatively however, we have not noted any recurrence in our patients with this method. This degree of lengthening agrees reasonably well with that of a previous clinical evaluation on the efficacy of the single square flap method in joint contracture reconstruction [11].

Given the risk of recurrence of axillary contractures in general, accompanying joint stiffness, and challenges with splinting, surgery alone is not sufficient to restore complete shoulder range of motion. We therefore employ a multi-modal management strategy to help restore functional capacity of the axilla and shoulder joint. Regarding rehabilitation and physiotherapy, it begins even prior to suture removal at approximately one week postoperatively. In the early phases of this program, we emphasize continuous passive ranging exercises to stretch the ligaments and muscles which have likely been shortened over prolonged periods of time. The patient is then transitioned to active range along with strengthening exercises, to recondition atrophied and unused muscles. A focus on returning to regular activities of daily living is maintained throughout rehabilitation, with an additional goal of restoring function that may have been lost since the initial burn injury. 

Despite expanding on Kurtzman’s system over 20 years ago, and continued experience in the area, reconstruction of the contracted axilla remains a complex problem. Its intricate anatomical structure, and key functional demands contribute to this challenge. However, relatively simple techniques such as the square flap method, and two facing square flaps remain powerful. When applied appropriately, in the carefully selected patient, form and function can be restored, and pleasing results can be achieved.

## 5. Conclusions

The two facing square flaps method is a comparatively simple reconstructive strategy for axillary contractures involving both the anterior and posterior lines, and should be within armamentarium of most plastic surgeons. Although the axilla remains difficult to reconstruct in burn patients, a favourable 3–4 times lengthening can be achieved with this method, and contracture recurrence is low in the appropriately selected patient.

## Data Availability

No new data were created or analyzed in this study. Data sharing is not applicable to this article.
